# Physical Function in Amateur Athletes with Lumbar Disc Herniation and Chronic Low Back Pain: A Case-Control Study

**DOI:** 10.3390/ijerph19063743

**Published:** 2022-03-21

**Authors:** Diego Miñambres-Martín, Patricia Martín-Casas, Ibai López-de-Uralde-Villanueva, César Fernández-de-las-Peñas, Juan Antonio Valera-Calero, Gustavo Plaza-Manzano

**Affiliations:** 1 Premium Madrid Global Health Care, 28016 Madrid, Spain; diego.minambres@rehabilitacionpremiummadrid.com; 2 Faculty of Sport Sciences, Universidad Europea de Madrid, 28670 Villaviciosa de Odón, Spain; 3 Department of Radiology, Rehabilitation and Physiotherapy, Universidad Complutense de Madrid, 28040 Madrid, Spain; pmcasas@enf.ucm.es (P.M.-C.); ibailope@ucm.es (I.L.-d.-U.-V.); gusplaza@ucm.es (G.P.-M.); 4 Instituto de Investigación Sanitaria San Carlos (IdISSC), 28040 Madrid, Spain; 5 Department of Physical Therapy, Occupational Therapy, Rehabilitation and Physical Medicine, Universidad Rey Juan Carlos, 28922 Alcorcón, Spain; cesar.fernandez@urjc.es; 6 Cátedra Institucional en Docencia, Clínica e Investigación en Fisioterapia: Terapia Manual, Punción Seca y Ejercicio Terapéutico, Universidad Rey Juan Carlos, 28922 Alcorcón, Spain; 7 VALTRADOFI Research Group, Department of Physiotherapy, Faculty of Health, Universidad Camilo José Cela, 28692 Villanueva de la Cañada, Spain

**Keywords:** low back pain, lumbar disc herniation, physical activity, muscle function, range of movement

## Abstract

This study aimed to analyze if chronic low back pain (LBP) and lumbar disc herniation induce biomechanics, flexibility, body balance, physical activity, and muscular function alterations compared to a similar asymptomatic cohort. Fifty male volunteers (*n* = 25 with chronic LBP and lumbar disc herniation and *n* = 25 pain-free subjects) were enrolled. Range of motion (internal and external hip rotation, ankle dorsiflexion, and active straight leg raise, ASLR), trunk flexibility (finger–floor distance), body balance (Y-balance test) and muscle function (Biering–Sorensen test, prone and lateral bridges) outcomes were assessed. Comparative analyses between sides and group were conducted. Results: Patients showed greater weight and BMI compared with controls (*p* < 0.05). None of the outcomes bilaterally assessed showed side-to-side differences for pain-free participants (all, *p* > 0.05) or LBP patients (all, *p* > 0.05). Regarding the differences between groups, LBP patients showed limited internal hip rotation (*p* < 0.001), finger–floor distance (*p* < 0.001), body balance (*p* < 0.001), and muscle endurance (planks *p* < 0.001; Biering–Sorensen test *p* < 0.05). External hip rotation, ASLR, and ankle dorsiflexion range of movement were comparable in both groups (*p* > 0.05). The sample of pain-free amateur athletes showed greater range of movement for internal hip rotation, lower finger–floor distance, better body balance, and muscle function. However, the external hip rotation, ankle dorsiflexion, and ASLR tests showed no difference between cases and controls.

## 1. Introduction

Low back pain (LBP) is the most common musculoskeletal condition, affecting up to the 18.3% of the entire population with a lifetime prevalence of >80%, and is considered the largest contributor to years lived with disability by the Global Burden of Disease [1,2,3]. As the estimated cost per LBP episode and patient was estimated at EUR > 2750 considering direct and indirect costs (i.e., pharmaceuticals, surgical and non-surgical inpatient cares, medical visits, rehabilitation, and work absence), LBP represents a major health problem [4].

Since mechanical LBP refers to pain that arises intrinsically from spinal structures (e.g., intervertebral discs, sacroiliac joints, facet joints, bones, nerve roots, and muscles), different clinical classifications for LBP have been proposed, including lumbosacral muscle strain, disk herniation, lumbar spondylosis, spondylolisthesis, vertebral compression fractures, and traumatic injuries [5]. In patients with chronic low back pain, an estimated 39% of cases could be attributed to the intervertebral disk infection, torsion, or internal disk disruption even if not all degenerated or herniated disks are painful [6].

Functional testing is an essential procedure during the clinical evaluation, as the primary reason LBP populations seek medical care is difficulty performing regular functional activities [7]. In fact, some associations between mobility, strength and endurance of the trunk, balance, and sociodemographic features with future LBP have been found [8].

Although numerous previous studies have found significant associations between LBP and decreased ankle dorsiflexion [9], lumbar instability [10], hip range of movement [11], trunk flexibility [12], body balance [13], and function of lumbopelvic muscles [14], there is limited evidence comparing all these features in amateur athletes with disk herniation and chronic low back pain compared with a similar pain-free cohort. The rationale for this study was based on (1) the high LBP prevalence in amateur athletes (LBP point prevalence up to 67%, one-year prevalence up to 94%, and lifetime prevalence up to 84%, depending on the sport performance) [15] and (2) the lack of studies assessing functional and clinical testing in amateur athletes.

Therefore, the purpose of this study is to compare physical function factors considered to be associated with LBP (i.e., biomechanics, flexibility, body balance, physical activity, and muscular function) among the general population in one sample of amateur athletes with lumbar disk herniation and chronic low back pain with one sample of asymptomatic amateur athletes.

## 2. Materials and Methods

### 2.1. Study Design

An observational case–control study was conducted to compare the physical function among amateur athletes with chronic LBP and lumbar disk herniation and asymptomatic amateur athletes. This study followed the Strengthening the Reporting of Observational studies in Epidemiology (STROBE) guidelines and checklist for case–control studies [16]. Thus, the study design considered all the recommendations stated in the Declaration of Helsinki and approved by the local Clinical Ethics Committee of Hospital Clínico San Carlos (ID: 19/044-E_Tesis). All participants signed a written informed consent prior to their inclusion. Demographic data including age, gender, height, weight, and body mass index (BMI), and number of LBP episodes during the last 12 months were collected in a standard self-reported history template.

### 2.2. Participants

Two consecutive samples of individuals were recruited and screened for potential eligibility in a tertiary physical therapy clinic in Madrid (Spain) from September 2019 to March 2020. Local announcements were posted to inform potential participants during the recruitment stage. General inclusion criteria applicable for both samples were (1) adults aged ranging 25 to 55 years, since this is the peak range of lumbar disk herniation prevalence [17], and (2) rutinary physical activity performance (150 min/week at moderate intensity, 75 min/week at vigorous intensity, or equivalent combination of both). Exclusion criteria included (1) traumatic injury or surgery located in the lumbar spine or lower limbs during the last year; (2) local conditions affecting the functional tests performance; (3) subjects needing foot orthose; (4) upper or lower limbs asymmetries; (5) peripheral or central neuropathies; (6) cognitive, psychological, or psychiatric conditions; (7) any other inflammatory or malignancy medical condition.

Specific criteria for cases were (1) at least one relevant episode of LBP during the last year, (2) with a duration under 3 months, (3) without neurological signs, and (4) imaging confirmation of lumbar disk herniation. Controls had to confirm absence of LBP during the last year to be included in the study.

### 2.3. Clinical Severity Assessment

#### 2.3.1. Pain Intensity

Participants with chronic LBP and disc herniation were asked to rate, on a 11-point numerical point rate scale (NPRS), their mean pain intensity experienced during the last LBP episode. In this scale, 0 was interpreted as “complete absence of pain” and 10 as “the worst imaginable pain” [18].

#### 2.3.2. Low Back Pain Disability

In order to quantify the disability caused by LBP, participants were asked to complete the Oswestry Low Back Pain Disability Questionnaire (ODI). This self-reported questionnaire demonstrates acceptable reproducibility [19] and consists of a 10-item battery and responses in a 6-point Likert scale where 0 is considered “complete absence of disability” and 5 “maximum disability”. Final scores are expressed as a percentage and results from multiplying the test score times two. Participants’ disability is considered as minimal, moderate, severe, crippled, or complete if final results range from 0–20%, 21–40%, 41–60%, 61–80%, and 81–100%, respectively [20].

### 2.4. Physical Function Assessment

#### 2.4.1. Biomechanics

Passive ankle dorsiflexion was measured following the procedure described by Konor et al. and using a digital inclinometer as recommended [21]. Participants were placed in the standing position (allowing the contact with the wall using two fingers to ensure the balance) with the heel in contact with the ground, the knee in line with the second toe and the great toe 10 cm away from the wall. Then, participants were asked to lunge forward, directing their knees toward the wall until their knees touched the wall keeping the heel in the ground. This procedure was repeated, gliding the foot back (at a rate of 1 cm at a time) until finding the last point the participant could touch the wall with the knee. To obtain the range of movement (ROM), a digital inclinometer was placed at the anterior tuberosity of the tibia to measure the relative angle of the tibia to the ground.

In addition, internal and external passive hip rotation were assessed bilaterally using a digital inclinometer. Participants were placed in the prone position, knee flexed to 90° and pelvis stabilized using a belt. To improve the accuracy of measurements, a mean average of three trials was calculated for each movement and side, and finally analyzed [22].

#### 2.4.2. Flexibility

Lower limb and trunk flexibility were assessed using the ASLR and finger–floor distance, respectively. Participants were placed in a supine position with hips and knees extended at rest and feet 20 cm apart. A digital inclinometer was placed at the anterior tuberosity of the tibia. During the test, the examiner passively lifted the leg up, keeping the knee extension until the participant’s limit (defined and trained as first appearance of discomfort) to obtain the ROM [23].

Finger–floor distance was measured with participants in the standing position, hips in neutral rotation, knees extended, and feet separated 20 cm apart. Then, participants were asked to perform a maximum trunk flexion avoiding the knee flexion, keeping both hands touching and fingers extended. To obtain the score, the examiner measured the distance between the floor and the distal extreme of the third finger with a metric line [24].

#### 2.4.3. Physical Activity

Rutinary physical activity was measured using the Global Physical Activity Questionnaire (GPAQ) since it is considered as a reliable, valid, and adaptable self-reported questionnaire across all populations. This questionnaire consists of 16 questions within three domains: occupational, transport-related, and leisure-time physical activity that lasts for at least 10 min with the moderate and vigorous intensity [25].

#### 2.4.4. Balance

Balance was assessed using the Y-balance test. To complete the test, participants were standing at the center of a Y, with the hands in contact with the hips, and reached with the contralateral leg as far as possible along one of the three directions (anterior, posteromedial, and posterolateral) not touching the floor while maintaining a single-leg squat stance. Participants trained the test three times with a 30” period of resting to avoid fatigue before obtaining the score. The test was considered failed if the participant touched the ground with the foot during the maneuver, lost the balance, lost the contact of the hands with the hips, or glided the stance foot [26].

#### 2.4.5. Function of Trunk Muscles

Muscle function was assessed using the prone and lateral bridges and the Biering–Sorensen test. The prone and lateral bridge consists of the amount of time a participant can maintain a prone bridge (plank) and a side bridge (plank) on their feet, aligning shoulders, pelvis, and toes before losing the alignment [27].

Finally, the Biering–Sorensen test challenges the erector spinal endurance, assessing the amount of time a participant can keep the unsupported upper part of the body (from the iliac crest) horizontal while placed prone with the buttocks and legs fixed to a table and the arms across the chest until the participants lose control of the posture [28].

### 2.5. Statistical Analysis

Statistical analyses were performed with the SPSS V.25 software for Mac OS (IBM, Armonk, NY, USA). Normal distribution of the data was verified using the Shapiro–Wilk test. Sociodemographic, clinical, and functional characteristics were calculated by total sample, by sex and group (cases and controls). A two-way Analysis of Variance (ANOVA) with side as within-group factor (for bilaterally assessed variables) and group as between-group factor was used to determine side-to-side and between-group differences, respectively. All tests were two-tailed, with *p*-values < 0.05 considered significant.

## 3. Results

From 57 subjects who responded to the announcement, seven participants were excluded (1) from the control group because they reported history of LBP during within the last 6 months (*n* = 4), and (2) from the LBP group since they did not have imaging confirmation of disc herniation (*n* = 3). Therefore, fifty participants (*n* = 25 patients with chronic LBP and lumbar disc herniation and *n* = 25 pain-free controls, all males) were finally included.

Sociodemographic and clinical characteristics from both samples are described in Table 1. Between-sex differences could not be analyzed since all participants were males. Regarding the comparison between both samples, significant differences were found for weight and BMI (both, *p* < 0.05). In addition, pain-free subjects showed to be more active than patients (greater vigorous activity and less sitting time, both *p* < 0.001).

Range of movement and flexibility data are summarized in Table 2. None of the outcomes bilaterally assessed showed side-to-side differences for pain-free participants (all, *p* > 0.05) or LBP patients (all, *p* > 0.05). However, LBP patients showed significant internal hip rotation limitation compared to asymptomatic subjects (*p* < 0.001). External hip rotation, ASLR, and ankle dorsiflexion range of movement were comparable in both groups (*p* > 0.05). Finger–floor distance was significantly greater in patients with LBP (*p* < 0.001).

Balance scores by group and side are reported in Table 3. Asymptomatic and LBP populations showed no side-to-side differences for the Y-balance test (total score and all individual axes, *p* > 0.05). Although the anterior axis showed no differences between LBP and pain-free samples (*p* > 0.05), pain-free subjects resulted in greater scores in both posterior axes (posteromedial *p* < 0.001; posterolateral *p* < 0.01) and the total score (*p* < 0.001).

Finally, in Table 4, data regarding the muscular function in both samples are available. During the lateral bridge, both samples obtained comparable timings for the left and the right side (*p* > 0.05). However, prone and lateral bridge timings were significantly larger in the asymptomatic sample compared to the LBP group (both, *p* < 0.001). Regarding the Biering–Sorensen test, the pain-free group held the posture control significantly longer than LBP group (*p* < 0.05).

## 4. Discussion

Although this is not the first study investigating functional differences between patients with chronic LBP and lumbar disc herniation and asymptomatic subjects, to our knowledge, no previous reports are available assessing these differences in amateur athletes. In general, we found no side-to-side differences for bilaterally assessed tests in both the clinical and asymptomatic groups. Regarding the between-groups differences, pain-free subjects demonstrated better muscle function in all the tests (prone and lateral bridges and Biering–Sorensen test) and balance (except Y-balance test scores in the anteroposterior axis), even if disability scores and pain intensity of the symptomatic subjects were fair. However, not all the range of movement and flexibility tests showed relevant differences. For instance, ankle dorsiflexion, the ASLR test, and external hip rotation scores were comparable between groups. Both internal hip rotation and finger–floor distance were clinically relevant to distinguish clinical and asymptomatic subjects.

Measuring the range of motion is a common clinical practice aiming at identifying dysfunctional patterns and comparing the patients’ evolution [29]. A previous systematic review reported that general LBP populations are characterized by decreased ROM compared to asymptomatic subjects. In addition, normative lumbar ROM scores for asymptomatic subjects are reported. However, these scores showed wide ranges due to variations between studies (i.e., including biological conditions, measuring instruments, and positions) and the clinical relevance is limited [30].

Regarding the hip joint, the reduced hip extension in patients with LBP and compensatory lumbar spine rotation during hip movements are consistent in the literature [31,32,33,34]. Thus, this hip extension limitation is directly associated with greater hip extension asymmetry, pain intensity, and disability in general LBP populations. In accordance with our findings, internal hip rotation is reduced in amateur golfers with LBP, professional tennis players, and sedentary subjects compared to asymptomatic controls [33]. However, we did not find asymmetric range of motion as did Kim et al. [33]. This inconsistence can be explained by the fact that we did not focus on specific sport performance, and the asymmetric nature of some sports (e.g., golf or tennis) with repeated pivoting movements (e.g., tennis and golf) causing high torsional forces on the lead hip could be the main reason for asymmetric hip range of motion.

The utility of the ASLR remains controversial since its diagnostic accuracy for identifying sciatica showed a 36–79% sensitivity and 37–74% specificity and, therefore, there is low level of evidence supporting the use of ASLR [35]. Accordingly, we found no differences between groups or sides.

On the other hand, our results showed relevant finger–floor distance differences between both groups. Similarly, a previous study conducted a cluster of eight signs (including this test in the battery) for diagnosing lumbosacral nerve root compression, demonstrating acceptable sensitivity (72%) and specificity (80%) [36].

In this study, we found LBP patients to obtain poorer balance scores compared with pain-free subjects, especially in the posterolateral and posteromedial directions. Similarly, Ganesh et al. [37] obtained similar conclusions, as LBP subjects reached shorter distances than asymptomatic subjects. However, our results cannot be compared, since sociodemographic characteristics of the participants are not described in the study conducted by Ganesh et al. [37].

Greater muscle function was found in pain-free controls compared with LBP patients. Emphasizing core endurance is essential, as poor endurance and motor control strategies have been widely associated with LBP [38,39]. In contrast to the conclusions made by Pitcher et al. [40], where the Biering–Sorensen test is not conclusive enough to differentiate between controls and subjects with varying degrees of mild back disability based on the Oswestry classification, we found significant differences between amateur athletes with mild disability and asymptomatic athletes. However, these tests are highly demanding, and better-tolerated alternatives (i.e., isometric and dynamic exercises such as squat or bird-dog exercises) should be considered during rehabilitative exercise programs [41].

### Limitations

Some potential limitations should be recognized for the current study. Given that only men were included, these findings may not be applicable to women with this condition. In addition, as asymptomatic volunteers were not required to present any imaging test, we cannot confirm the absence of lumbar disc herniation or any other radiological finding for this group. Finally, the sample size of this study was too small to be able to extrapolate our findings to the general population. Further research increasing the sample size would increase the statistical power of the study to corroborate/deny our findings. Despite these potential limitations, current findings could assist clinicians targeting therapies for future clinical intervention trials in individuals with lumbar disc herniation and chronic low back pain.

## 5. Conclusions

Although our sample of amateur athletes with lumbar disc herniation and chronic LBP showed mild disability and pain, several functional differences with pain-free controls were found. The sample of asymptomatic subjects showed greater range of movement for internal hip rotation, lower finger–floor distance, better balance, and muscle function. However, the external hip rotation, ankle dorsiflexion, and ASLR tests showed no difference between cases and controls.

## Figures and Tables

**Table 1:** Baseline characteristics of the sample by group.

	Total Sample (*n* = 50)	LBP Group (*n* = 25)	Pain-Free Group (*n* = 25)
*Socio-demographic characteristics*			
Gender (*n*, H/M)	50/0	25/0	25/0
Age (years)	37.1 ± 6.6	38.3 ± 7.9	35.9 ± 4.8
Height (meters)	1.75 ± 0.07	1.76 ± 0.08	1.75 ± 0.05
Weight (kg) *	75.9 ± 11.9	79.2 ± 14.4	72.6 ± 7.8
BMI (kg/m^2^) *	24.4 ± 2.5	25.2 ± 2.8	23.6 ± 1.8
*Clinical Characteristics*			
NPRS (0–100)		3.8 ± 3.8	N/A
LBP Episodes (*n*) ^a^		2.0 ± 2.11	N/A
ODI (0–100)		6.3 ± 8.7	N/A
*Physical Activity*			
Vigorous Activity—Sports (METS) ^†^	1826.4 ± 486.0	1320.0 ± 382.2	2332.8 ± 385.1
Moderate Activity—Sports (METS)	609.6 ± 161.5	751.2 ± 192.9	468.0 ± 173.2
+10 Walking (METS)	416.4 ± 112.7	393.6 ± 104.7	439.2 ± 109.3
Sitting time (hours/day) ^†^	9.14 ± 3.70	11.4 ± 2.2	6.8 ± 3.4

Values are expressed as mean ± SD. ^a^ Number of LBP episodes within the last year. * Significant differences between case and control groups (*p* < 0.05). ^†^ Significant differences between case and control groups (*p* < 0.001).

**Table 2:** Participants’ characteristics at baseline.

	Internal Hip Rotation (°)	External Hip Rotation (°)	ASLR (°)	Ankle Dorsiflexion (°)	Finger–Floor Distance (cm)
*LBP Group (n = 25)*					
Mean	14.2 ± 4.3	27.3 ± 7.1	58.1 ± 9.8	42.8 ± 9.6	8.8 ± 9.9
Left side	13.8 ± 4.5	27.2 ± 5.8	58.4 ± 9.3	41.1 ± 8.1	
Right side	14.5 ± 4.2	27.3 ± 6.4	57.7 ± 10.4	40.8 ± 7.2	
Between-side differences	0.6 (−1.8; 3.1)	0.0 (−3.6; 3.7)	0.7 (−1.8; 3.2)	0.3 (−4.0; 4.7)	
*Pain-free Group (n = 25)*					
Mean	17.3 ± 5.9	27.1 ± 5.3	60.8 ± 9.0	42.8 ± 9.6	−0.6 ± 6.22
Left side	16.9 ± 6.2	27.6 ± 5.6	60.6 ± 8.7	42.5 ± 8.7	
Right side	17.7 ± 5.7	26.7 ± 4.9	61.1 ± 9.4	43.2 ± 10.7	
Between-side Difference	0.8 (−2.6; 4.2)	0.9 (−2.1; 3.9)	0.5 (−4.7; 5.7)	0.6 (−4.9; 6.2)	
Between-Group Differences					
Mean	3.1 (−1.0; 5.2) *	0.1 (−2.2; 2.4)	2.7 (−6.5; 0.9)	1.8 (−1.5; 5.3)	9.4 (4.6; 14.1) *

* Statistically significant differences (*p* < 0.001).

**Table 3:** Participants characteristics at baseline.

	Y-Balance Test Total Score (°)	Y-Balance Test Anterior Axis (°)	Y-Balance Test Posteromedial Axis (°)	Y-Balance Test Posterolateral Axis (°)
*LBP Group (n = 25)*				
Mean	184.6 ± 25.0	54.4 ± 6.4	62.1 ± 11.1	68.0 ± 11.7
Left side	185.4 ± 25.5	54.4 ± 6.5	63.2 ± 10.5	67.8 ± 12.0
Right side	183.8 ± 25.4	54.4 ± 6.5	61.0 ± 11.8	68.3 ± 11.6
Between-side differences	1.5 (−12.8; 15.9)	0.0 (−3.6; 3.7)	2.1 (−4.2; 8.5)	0.6 (−6.1; 7.3)
*Pain-free Group (n = 25)*				
Mean	200.2 ± 18.0	55.8 ± 5.4	70.5 ± 7.9	73.8 ± 8.4
Left side	199.0 ± 18.6	55.7 ± 5.7	69.2 ± 8.3	74.0 ± 8.2
Right side	201.4 ± 17.7	55.8 ± 5.3	71.9 ± 7.4	73.6 ± 8.7
Between-side Difference	2.4 (−7.9; 12.8)	0.0 (−3.1; 3.2)	2.7 (−1.8; 7.2)	0.4 (−4.4; 5.2)
*Between-Group Differences*				
Mean	15.5 (6.8; 24.2) ^†^	1.3 (−1.0; 3.7)	8.4 (4.5; 12.2) ^†^	5.7 (1.7; 9.8) *

* *p* < 0.01; ^†^ *p* < 0.001.

**Table 4:** Physical function characteristics: muscle function.

	LBP Group (*n* = 25)	Pain-Free Group (*n* = 25)	Between-Groups Differences
Prone Bridge (s)	25.9 ± 8.6	38.7 ± 14.4	12.8 (6.0; 17.0) ^†^
Lateral Bridge (s)			
*Mean*	19.4 ± 6.4	27.1 ± 9.7	7.7 (4.4; 11.0) ^†^
*Left side*	19.0 ± 5.7	26.0 ± 8.9	
*Right side*	19.8 ± 7.1	28.3 ± 10.4	
*Between-sides Difference*	0.7 (−3.0; 4.4)	2.2 (−3.2; 7.8)	
Biering-Sorensen Test (s)	29.1 ± 17.5	37.3 ± 13.1	8.2 (0.5; 17.0) *

* *p* <0.05; ^†^ *p* < 0.001.

## Data Availability

All data derived from this study are presented in the text.

## References

[1] Urits I., Burshtein A., Sharma M., Testa L., Gold P.A., Orhurhu V., Viswanath O., Jones M., Sidransky M.A., Spektor B. (2019). Low Back Pain, a Comprehensive Review: Pathophysiology, Diagnosis, and Treatment. Curr. Pain Headache Rep..

[2] Knezevic N.N., Candido K.D., Vlaeyen J.W.S., Van Zundert J., Cohen S.P. (2021). Low back pain. Lancet.

[3] Hoy D., March L., Brooks P., Blyth F., Woolf A., Bain C., Williams G., Smith E., Vos T., Barendregt J. (2014). The global burden of low back pain: Estimates from the Global Burden of Disease 2010 study. Ann. Rheum. Dis..

[4] Olafsson G., Jonsson E., Fritzell P., Hägg O., Borgström F. (2018). Cost of low back pain: Results from a national register study in Sweden. Eur. Spine J..

[5] Will J.S., Bury D.C., Miller J.A. (2018). Mechanical Low Back Pain. Am. Fam. Phys..

[6] Simon J., McAuliffe M., Shamim F., Vuong N., Tahaei A. (2014). Discogenic low back pain. Phys. Med. Rehabil. Clin. N. Am..

[7] Marich A.V., Hwang C.T., Sorensen C.J., van Dillen L.R. (2020). Examination of the Lumbar Movement Pattern during a Clinical Test and a Functional Activity Test in People with and without Low Back Pain. PM&R..

[8] Takala E.P., Viikari-Juntura E. (2000). Do functional tests predict low back pain?. Spine.

[9] Brantingham J.W., Lee Gilbert J., Shaik J., Globe G. (2006). Sagittal plane blockage of the foot, ankle and hallux and foot alignment-prevalence and association with low back pain. J. Chiropr. Med..

[10] Ferrari S., Manni T., Bonetti F., Villafañe J.H., Vanti C. (2015). A literature review of clinical tests for lumbar instability in low back pain: Validity and applicability in clinical practice. Chiropr. Man. Ther..

[11] Avman M.A., Osmotherly P.G., Snodgrass S., Rivett D.A. (2019). Is there an association between hip range of motion and nonspecific low back pain? A systematic review. Musculoskelet. Sci. Pract..

[12] Kemmochi M., Sasaki S., Ichimura S. (2018). Association between reduced trunk flexibility in children and lumbar stress fractures. J. Orthop..

[13] Kim D.H., Park J.K., Jeong M.K. (2014). Influences of posterior-located center of gravity on lumbar extension strength, balance, and lumbar lordosis in chronic low back pain. J. Back Musculoskelet. Rehabil..

[14] Smith B.E., Littlewood C., May S. (2014). An update of stabilisation exercises for low back pain: A systematic review with meta-analysis. BMC Musculoskelet. Disord..

[15] Farahbakhsh F., Rostami M., Noormohammadpour P., Mehraki Zade A., Hassanmirazaei B., Faghih Jouibari M., Kordi R., Kennedy D.J. (2018). Prevalence of low back pain among athletes: A systematic review. J. Back Musculoskelet. Rehabil..

[16] Cuschieri S. (2019). The STROBE guidelines. Saudi J. Anaesth..

[17] Kreiner D., Hwang S., Easa J., Resnick D., Baisden J., Bess S. (2014). An evidence-based clinical guideline for the diagnosis and treatment of lumbar disc herniation with radiculopathy. Spine J..

[18] Cheatham S.W., Kolber M.J., Mokha M., Hanney W.J. (2018). Concurrent validity of pain scales in individuals with myofascial pain and fibromyalgia. J. Bodyw. Mov. Ther..

[19] Garg A., Pathak H., Churyukanov M.V., Uppin R.B., Slobodin T.M. (2020). Low back pain: Critical assessment of various scales. Eur. Spine J..

[20] Fairbank J.C., Pynsent P.B. (2000). The Oswestry Disability Index. Spine.

[21] Konor M.M., Morton S., Eckerson J.M., Grindstaff T.L. (2012). Reliability of three measures of ankle dorsiflexion range of motion. Int. J. Sports Phys. Ther..

[22] Harris-Hayes M., Sahrmann S.A., Van Dillen L.R. (2009). Relationship between the hip and low back pain in athletes who participate in rotation-related sports. J. Sport Rehabil..

[23] Ekedahl K., Jönsson B., Frobell R. (2010). Validity of the fingertip-to-floor test and straight leg raising test in patients with acute and subacute low back pain: A comparison by sex and radicular pain. Arch. Phys. Med. Rehabil..

[24] Test F., Perret C., Poiraudeau S., Fermanian J., Anne M., Benhamou M. (2001). Validity, reliability, and responsiveness of the fingertip-to-floor test. Arch. Phys. Med. Rehabil..

[25] Keating X.D., Zhou K., Liu X., Hodges M., Liu J., Guan J., Phelps A., Castro-Piñero J. (2019). Reliability and Concurrent Validity of Global Physical Activity Questionnaire (GPAQ): A Systematic Review. Int. J. Environ. Res. Public Health..

[26] Powden C.J., Dodds T.K., Gabriel E.H. (2019). The reliability of the star excursion balance test and lower quarter y-balance test in healthy adults: A systematic review. Int. J. Sports Phys. Ther..

[27] Escamilla R.F., Lewis C., Pecson A., Imamura R., Andrews J.R. (2016). Muscle Activation Among Supine, Prone, and Side Position Exercises with and Without a Swiss Ball. Sports Health..

[28] Martínez-Romero M.T., Ayala F., Croix M.D.S., Vera-Garcia F.J., De Baranda P.S., Santonja-Medina F., Sánchez-Meca J. (2020). A Meta-Analysis of the Reliability of Four Field-Based Trunk Extension Endurance Tests. Int. J. Environ. Res. Public Health.

[29] Fourney D.R., Dettori J.R., Hall H., Härtl R., McGirt M.J., Daubs M.D. (2011). A systematic review of clinical pathways for lower back pain and introduction of the Saskatchewan Spine Pathway. Spine.

[30] Laird R.A., Gilbert J., Kent P., Keating J.L. (2014). Comparing lumbo-pelvic kinematics in people with and without back pain: A systematic review and meta-analysis. BMC Musculoskelet. Disord..

[31] Roach S.M., Juan J.G.S., Suprak D.N., Lyda M., Bies A.J., Boydston C.R. (2015). Passive hip range of motion is reduced in active subjects with chronic low back pain compared to controls. Int. J. Sports Phys. Ther..

[32] Sadeghisani M., Sobhani V., Kouchaki E., Bayati A., Ashari A.A., Mousavi M. (2015). Comparison of lumbopelvic and hip movement patterns during passive hip external rotation in two groups of low back pain patients with and without rotational demand activities. Ortop. Traumatol. Rehabil..

[33] Kim W.D., Shin D. (2020). Correlations Between Hip Extension Range of Motion, Hip Extension Asymmetry, and Compensatory Lumbar Movement in Patients with Nonspecific Chronic Low Back Pain. Med. Sci. Monit..

[34] Cejudo A., Moreno-Alcaraz V.J., Izzo R., Santonja-Medina F., Sainz de Baranda P. (2020). External and Total Hip Rotation Ranges of Motion Predispose to Low Back Pain in Elite Spanish Inline Hockey Players. Int. J. Environ. Res. Public Health.

[35] Mistry J., Heneghan N.R., Noblet T., Falla D., Rushton A. (2020). Diagnostic utility of patient history, clinical examination and screening tool data to identify neuropathic pain in low back related leg pain: A systematic review and narrative synthesis. BMC Musculoskelet. Disord..

[36] Vroomen P., de Krom M.C.T.F.M., Wilmink J., Kester A., Knottnerus J. (2002). Diagnostic value of history and physical examination in patients suspected of lumbosacral nerve root compression. J. Neurol. Neurosurg. Psychiatry.

[37] Ganesh G., Chhabra D., Mrityunjay K. (2015). Efficacy of the star excursion balance test in detecting reach deficits in subjects with chronic low back pain. Physiother. Res. Int..

[38] Abdelraouf O.R., Abdel-Aziem A.A. (2016). The relationship between core endurance and back dysfunction in collegiate male athletes with and without nonspecific low back pain. Int. J. Sports Phys. Ther..

[39] Lee J., Yoon C., Kim K., Cho M., Kim H.C., Chung S.G. (2019). Lumbar Stability in Healthy Individuals and Low Back Pain Patients Quantified by Wall Plank-and-Roll Test. PM&R..

[40] Pitcher M.J., Behm D.G., Mackinnon S.N. (2007). Neuromuscular fatigue during a modified biering-sørensen test in subjects with and without low back pain. J. Sports Sci. Med..

[41] Calatayud J., Escriche-Escuder A., Cruz-Montecinos C., Andersen L.L., Pérez-Alenda S., Aiguadé R., Casaña J. (2019). Tolerability and Muscle Activity of Core Muscle Exercises in Chronic Low-back Pain. Int. J. Environ. Res. Public Health.

